# Exploratory Pharmacokinetic Characterization of Enrofloxacin and Ciprofloxacin in Plasma and Interstitial Fluid of Dogs: Nonlinear Mixed-Effects Modeling and PK/PD Target Attainment Analysis

**DOI:** 10.3390/antibiotics15080716

**Published:** 2026-07-23

**Authors:** Patrik Mag, Zoltán Somogyi, Andrea Kertenics, Márton Kovács, Pál Szabó, Ákos Jerzsele

**Affiliations:** 1 Department of Pharmacology and Toxicology, University of Veterinary Medicine Budapest, H-1078 Budapest, Hungary; somzol92@gmail.com (Z.S.); jerzsele.akos@univet.hu (Á.J.); 2 National Laboratory of Infectious Animal Diseases, Antimicrobial Resistance, Veterinary Public Health and Food Chain Safety, University of Veterinary Medicine Budapest, H-1078 Budapest, Hungary; 3 Aurigon Labs Ltd., H-2120 Dunakeszi, Hungary; andrea.kertenics@aurigon.eu; 4 Research Center for Natural Sciences, Center for Structural Study, MS Metabolomics Laboratory, H-1117 Budapest, Hungary; marton.kovacs@ttk.hu (M.K.); szabo.pal@ttk.mta.hu (P.S.)

**Keywords:** enrofloxacin, ciprofloxacin, interstitial fluid, ultrafiltration, population pharmacokinetics, PK/PD, probability of target attainment, Monte Carlo simulation, dogs, fluoroquinolones

## Abstract

Background: Assessment of antimicrobial exposure at the site of infection is essential for pharmacokinetic/pharmacodynamic (PK/PD)-guided antimicrobial therapy, yet plasma concentrations may not adequately reflect extracellular target-site exposure. This study characterized the pharmacokinetics of enrofloxacin and its active metabolite ciprofloxacin in plasma and interstitial fluid (ISF) following high-dose oral enrofloxacin administration in dogs and evaluated PK/PD target attainment using nonlinear mixed-effects (NLME) modeling and Monte Carlo simulation. Methods: Nine healthy Beagle dogs were enrolled and received a single oral dose of enrofloxacin (target dose 20 mg/kg). Following exclusion of one dog after vomiting, pharmacokinetic analyses included plasma data from eight dogs and interstitial fluid (ISF) data from six dogs. Serial plasma samples and protein-free ISF samples collected over 24 h by in vivo ultrafiltration were analyzed using LC–MS/MS. Non-compartmental and population pharmacokinetic analyses were performed separately for plasma and ISF data. Final population models were used to simulate 5000 virtual individuals for probability of target attainment (PTA) analyses across a range of minimum inhibitory concentrations (MICs) using established AUC_0–24_/MIC and C_max_/MIC targets. Results: Both enrofloxacin and ciprofloxacin exhibited delayed peak concentrations and greater overall exposure in ISF than in plasma. For enrofloxacin, mean AUC_0–24_ was higher in ISF than in plasma (44.9 vs. 21.6 µg × h/mL). Exploratory population pharmacokinetic models adequately described the plasma and ISF concentration-time data. Monte Carlo simulations showed consistently higher PTA in ISF than in plasma. For the more conservative AUC_0–24_/MIC targets (≥100 and ≥125), the MIC associated with ≥90% PTA for enrofloxacin was four-fold higher in ISF than in plasma (0.125 vs. 0.03 µg/mL). Conclusions: High-dose oral enrofloxacin produced sustained extracellular exposure, resulting in more favorable PK/PD target attainment in ISF than in plasma. These findings indicate that plasma pharmacokinetics alone may not accurately reflect target-site antimicrobial exposure and support further evaluation of ISF-based pharmacokinetic data for PK/PD-guided optimization of fluoroquinolone therapy in dogs.

## 1. Introduction

Antimicrobial resistance (AMR) is a major concern in both human and veterinary medicine and has increased the importance of antimicrobial stewardship and prudent antimicrobial use [1,2]. In veterinary medicine, optimization of antimicrobial therapy based on pharmacokinetic/pharmacodynamic (PK/PD) principles is increasingly applied to maximize therapeutic efficacy while minimizing selection pressure for antimicrobial resistance [3]. Fluoroquinolones are considered critically important antimicrobials because of their broad-spectrum activity and their relevance in human medicine. Consequently, their veterinary use is subject to increasing regulatory scrutiny [4]. The European Medicines Agency (EMA), through the Antimicrobial Advice Ad Hoc Expert Group (AMEG), classified fluoroquinolones into Category B (“Restrict”), indicating that these compounds should only be used when no lower-risk antimicrobial alternatives are expected to be clinically effective [5].

Enrofloxacin is an established veterinary fluoroquinolone with favorable oral bioavailability and extensive tissue distribution in dogs [6]. Like other fluoroquinolones, it exhibits concentration-dependent antibacterial activity, and both the ratio of the area under the concentration-time curve to the minimum inhibitory concentration (AUC/MIC) and the ratio of peak concentration to MIC (C_max_/MIC) have been identified as major predictors of antibacterial efficacy [3,7,8]. The minimum inhibitory concentration (MIC) serves as the critical link between antimicrobial exposure and bacterial susceptibility, making accurate characterization of drug exposure an essential component of PK/PD-guided antimicrobial therapy [9,10].

Enrofloxacin undergoes partial hepatic metabolism to ciprofloxacin in dogs [6,11]. Ciprofloxacin is an active fluoroquinolone metabolite and may contribute to the overall antibacterial activity following enrofloxacin administration [6,11]. Consequently, simultaneous evaluation of both compounds provides a more complete characterization of fluoroquinolone exposure than assessment of the parent compound alone.

Several studies have investigated the pharmacokinetics of enrofloxacin and ciprofloxacin following oral administration in canine plasma [11,12,13,14,15]. In contrast, information regarding extracellular drug exposure remains considerably more limited. To date, only two studies have characterized enrofloxacin disposition in canine interstitial fluid (ISF) using ultrafiltration techniques [11,14]. These investigations demonstrated the feasibility of repeated ISF sampling and provided important information regarding extracellular drug distribution. However, both studies evaluated conventional enrofloxacin dosage regimens and relied primarily on non-compartmental pharmacokinetic analyses. To the authors’ knowledge, plasma and ISF pharmacokinetics following administration of high-dose oral enrofloxacin (20 mg/kg) have not been previously characterized simultaneously in dogs, nor have they been evaluated using a combined population pharmacokinetic modeling and probability of target attainment (PTA) approach.

Assessment of antimicrobial concentrations at the site of infection has become an increasingly important component of PK/PD-guided antimicrobial therapy [3]. Plasma concentrations do not necessarily reflect free antimicrobial concentrations within peripheral tissues, particularly within extracellular compartments where many bacterial infections develop [16]. Consequently, reliance on plasma pharmacokinetics alone may underestimate or misrepresent antimicrobial exposure at the pharmacological target site. Interstitial fluid is generally considered representative of extracellular free drug concentrations and therefore provides clinically relevant information regarding tissue exposure [16,17]. Ultrafiltration probe techniques enable repeated minimally invasive ISF sampling and have been successfully applied in experimental canine studies [11,14]. Nevertheless, comparative evaluations of plasma- and ISF-based PK/PD target attainment remain scarce.

In the European Union, the authorized dose of enrofloxacin for dogs is generally 5 mg/kg once daily. Oral enrofloxacin doses up to 20 mg/kg have been approved by the United States Food and Drug Administration [18]. Therefore, the high-dose regimen investigated in the present study corresponds to the upper end of the FDA-approved dose range but exceeds the standard authorized European dose. Because fluoroquinolones exhibit concentration-dependent antibacterial activity, characterization of plasma and tissue exposure following high-dose administration is particularly relevant for evaluation of PK/PD target attainment across a range of MIC values. In addition, the investigation of extracellular exposure at higher dosage regimens may improve our understanding of the relationship between systemic and target-site pharmacokinetics [16].

Monte Carlo simulation and probability of target attainment (PTA) analyses are widely used tools in antimicrobial PK/PD research [7,19]. These approaches incorporate pharmacokinetic variability and allow estimation of the likelihood of achieving predefined PK/PD targets within a population. Although PTA analyses are increasingly applied in veterinary antimicrobial investigations [10,20], limited information is available regarding comparative PTA predictions derived from plasma and ISF exposure profiles in dogs.

Therefore, the aim of the present study was to characterize the pharmacokinetics of enrofloxacin and ciprofloxacin in plasma and interstitial fluid following administration of a single high oral dose of enrofloxacin in dogs, develop separate nonlinear mixed-effects (NLME) pharmacokinetic models for plasma and interstitial fluid data, and evaluate PK/PD target attainment using Monte Carlo simulation-based approaches.

## 2. Results

### 2.1. Data Availability and ISF Sampling

Nine dogs were enrolled in the study. One dog vomited approximately 20 min after enrofloxacin administration and was excluded from all subsequent analyses. This animal received the highest dose relative to body weight (225 mg; 24.2 mg/kg). Consequently, the final analytical dataset comprised eight dogs, in which the administered dose ranged from 15.3 to 21.0 mg/kg (mean: 19.0 mg/kg). As a result, plasma pharmacokinetic analyses included data from eight dogs, whereas ISF pharmacokinetic analyses included data from six dogs because ultrafiltration probe failure prevented ISF sample collection in two animals. Apart from one dog that vomited approximately 20 min after enrofloxacin administration and was excluded from further analyses, no clinically apparent adverse events were observed in the remaining animals during the 24 h observation period. The mean ISF flow rate across all evaluable ultrafiltration collections was 1.35 ± 0.60 µL/min (range 0.52–3.50 µL/min), resulting in an estimated lag time of 0.84 h. Flow rate measurements were used exclusively to estimate ultrafiltration lag time and were not applied to correct ISF drug concentrations.

### 2.2. Non-Compartmental Pharmacokinetic Analysis

**Figure 1:**
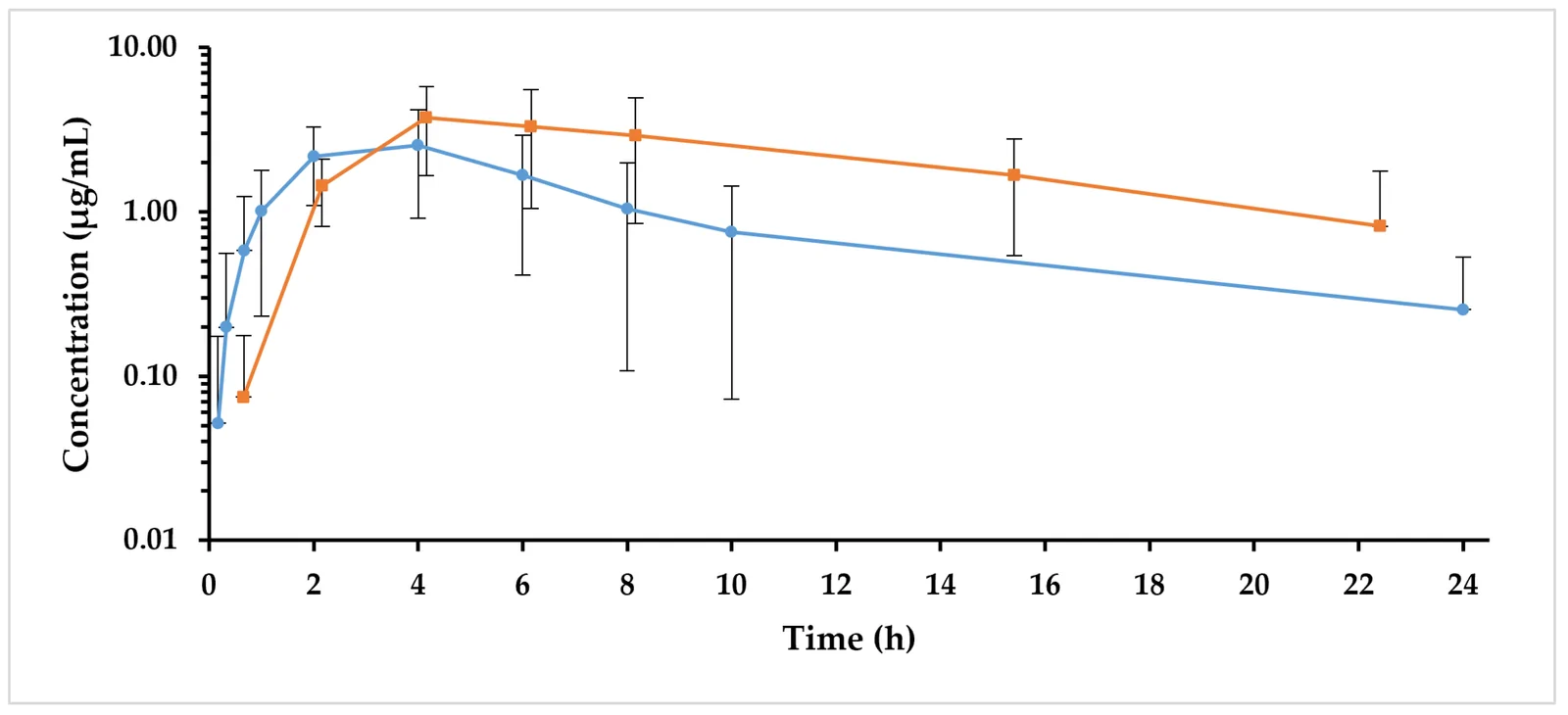
Plasma (blue circles) and interstitial fluid (ISF; orange squares) concentrations of enrofloxacin following a single oral administration of enrofloxacin tablets at a target dose of 20 mg/kg in dogs. Data are presented as mean ± SD. ISF sampling times were assigned to the midpoint of each collection interval and subsequently corrected for ultrafiltration lag time (t_UF_ = 0.84 h). The y-axis is presented on a logarithmic scale.

**Figure 2:**
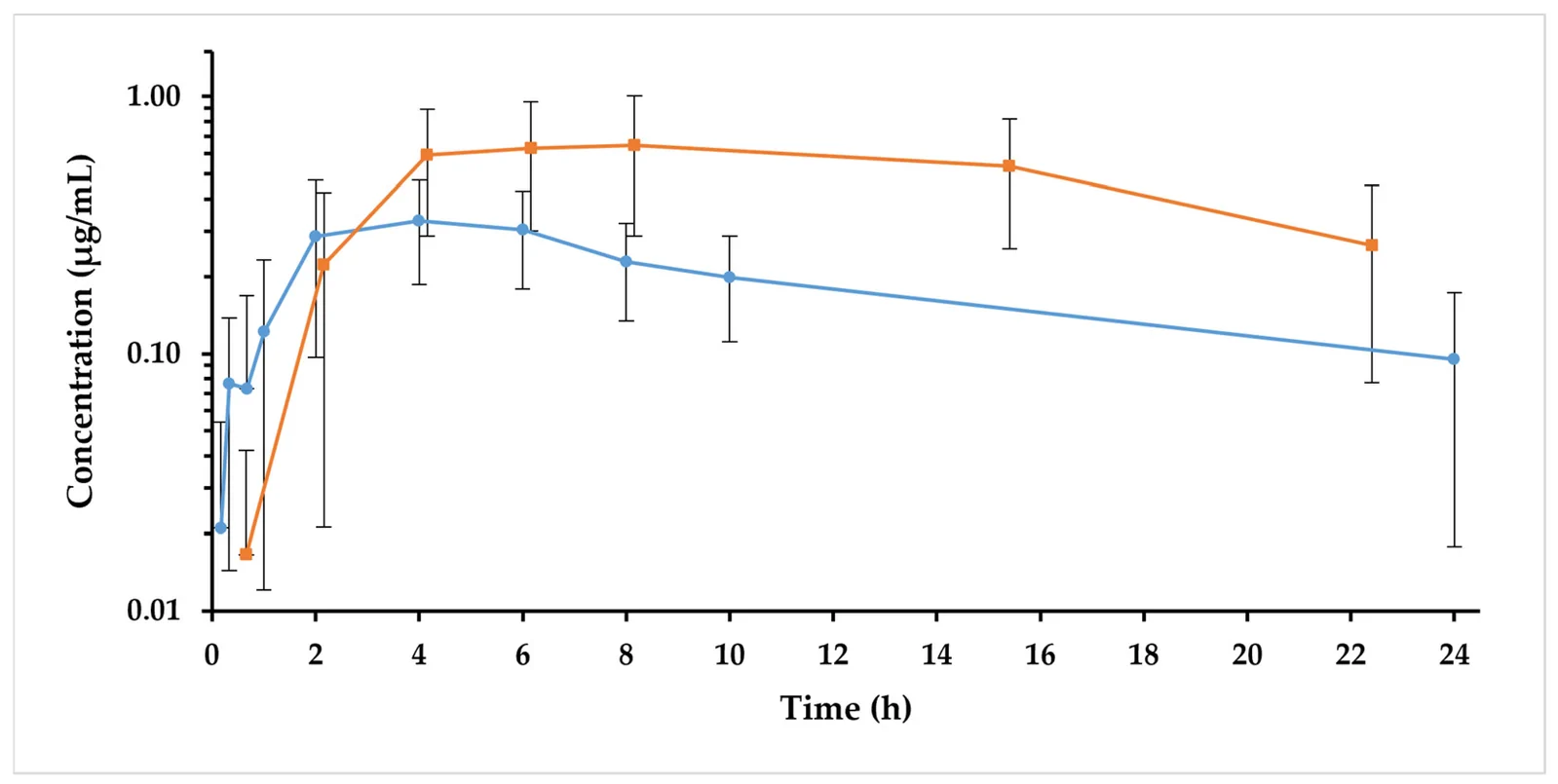
Plasma (blue circles) and interstitial fluid (ISF; orange squares) concentrations of ciprofloxacin following a single oral administration of enrofloxacin tablets at a target dose of 20 mg/kg in dogs. Data are presented as mean ± SD. ISF sampling times were assigned to the midpoint of each collection interval and subsequently corrected for ultrafiltration lag time (t_UF_ = 0.84 h). The y-axis is presented on a logarithmic scale.

Mean plasma and ISF concentration-time profiles of enrofloxacin and its metabolite ciprofloxacin following oral administration of enrofloxacin are shown in Figure 1 and Figure 2, respectively. For both compounds, ISF concentrations were generally higher than the corresponding plasma concentrations and attained maximal concentrations at later time points. Quantifiable ciprofloxacin concentrations were detected in both plasma and ISF throughout the 24 h observation period.

Non-compartmental pharmacokinetic parameters of enrofloxacin are summarized in Table 1. Mean maximum concentrations (C_max_) were 2.94 ± 1.39 µg/mL in plasma and 3.77 ± 2.07 µg/mL in ISF. Time to maximum concentration (T_max_) was significantly longer in ISF than in plasma. Exposure-related parameters (AU_C0–24_, AUC_inf_ and AUMC_inf_) were significantly higher in ISF than in plasma.

Non-compartmental pharmacokinetic parameters of ciprofloxacin are presented in Table 2. Mean maximum concentrations (C_max_) were 0.37 ± 0.15 µg/mL in plasma and 0.77 ± 0.34 µg/mL in ISF. Time to maximum concentration (T_max_) was significantly longer in ISF than in plasma. In addition, C_max_, AUC_0–24_ and AUC_inf_ were significantly higher in ISF, indicating greater ciprofloxacin exposure in the extracellular compartment. No significant differences were observed for λ_z_, t_1/2_, AUMC_inf_ or MRT_inf_.

### 2.3. Nonlinear Mixed-Effects Pharmacokinetic Modeling

All datasets were adequately described by one-compartment population pharmacokinetic models with first-order absorption, first-order elimination, and an absorption lag time. Final population pharmacokinetic parameter estimates are presented in Table 3, whereas estimates of interindividual variability (IIV) and η-shrinkage are summarized in Table 4.

The final models included the first-order absorption rate constant (K_a_), apparent volume of distribution (V/F), apparent clearance (CL/F), and absorption lag time (T_lag_) for all analyte-matrix combinations. Interindividual variability was retained on selected structural parameters depending on the analyte and sampling matrix. Variability was identified primarily on V/F and CL/F, with additional random effects retained on T_lag_ for enrofloxacin plasma and on K_a_ for ciprofloxacin ISF.

Different residual error structures were retained in the final models. Combined additive and multiplicative residual error models were applied to enrofloxacin plasma, enrofloxacin ISF, and ciprofloxacin ISF data, whereas an additive residual error model was retained for ciprofloxacin plasma concentrations. Estimated IIV ranged from moderate to high across the retained parameters, while η-shrinkage values remained low (Table 4).

Body weight was formally evaluated as a covariate on apparent clearance and apparent volume of distribution in each analyte–matrix model. Although inclusion of body weight resulted in minor numerical improvements in selected models, these effects were not consistent across analytes and matrices and were not uniformly supported by improvements in model diagnostics or numerical stability. Consequently, body weight was not retained in the final population pharmacokinetic models.

Observed and individual predicted concentrations were generally in good agreement for enrofloxacin and ciprofloxacin in both plasma and ISF. Most observations were distributed close to the line of identity in the DV versus IPRED plots, although greater dispersion around the identity line was evident for ciprofloxacin in plasma and at higher concentration ranges in some datasets.

**Figure 3:**
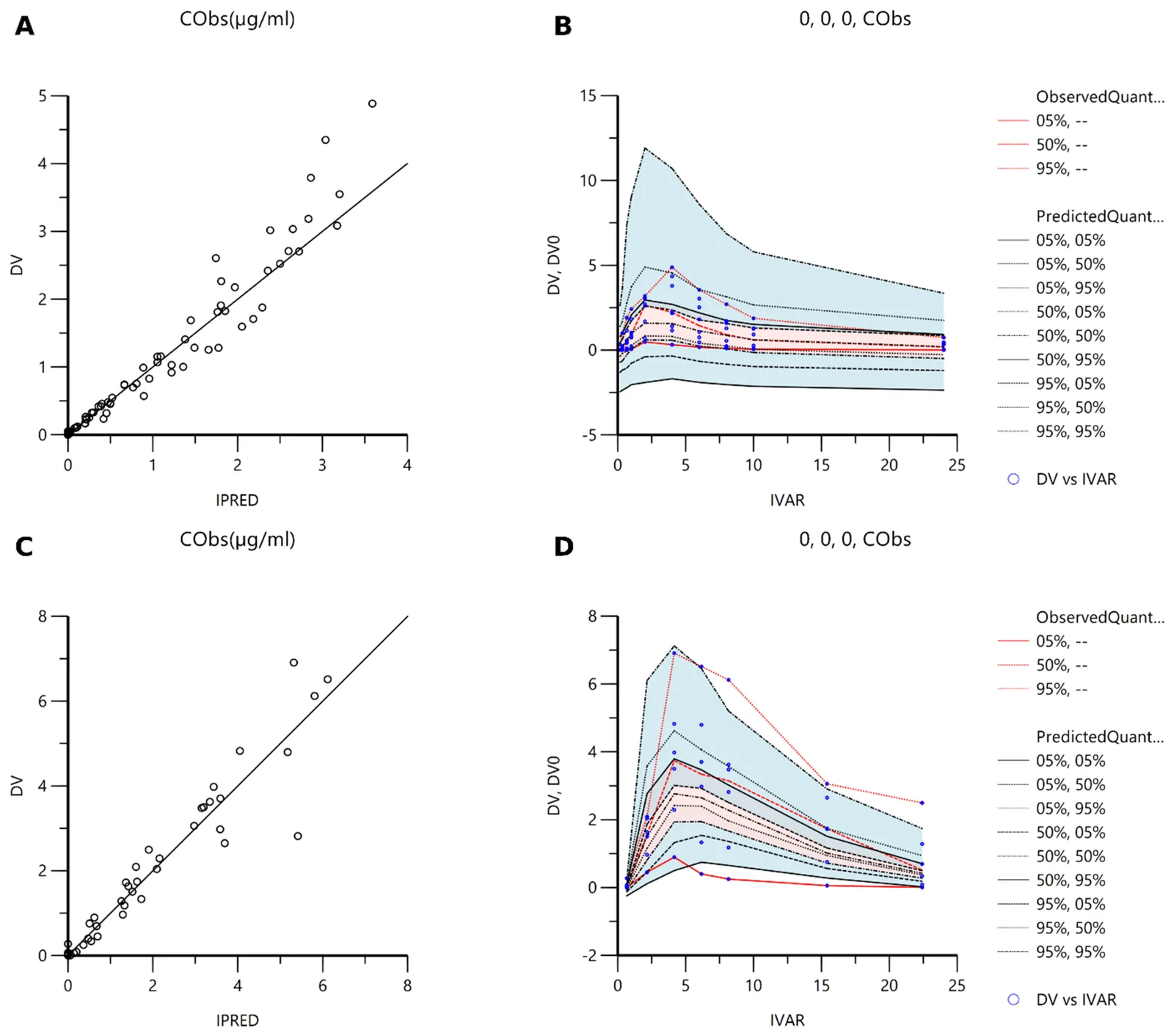
Goodness-of-fit diagnostics and visual predictive checks for the enrofloxacin population pharmacokinetic models. (**A**) Observed versus individual predicted enrofloxacin plasma concentrations. (**B**) Visual predictive check (VPC) for the enrofloxacin plasma model. (**C**) Observed versus individual predicted enrofloxacin interstitial fluid (ISF) concentrations. (**D**) Visual predictive check (VPC) for the enrofloxacin ISF model. Open circles represent observed concentrations. In the VPC plots, the red lines indicate the observed 5th, 50th, and 95th percentiles, while the black lines and shaded areas represent the corresponding simulated percentiles and their 95% confidence intervals.

**Figure 4:**
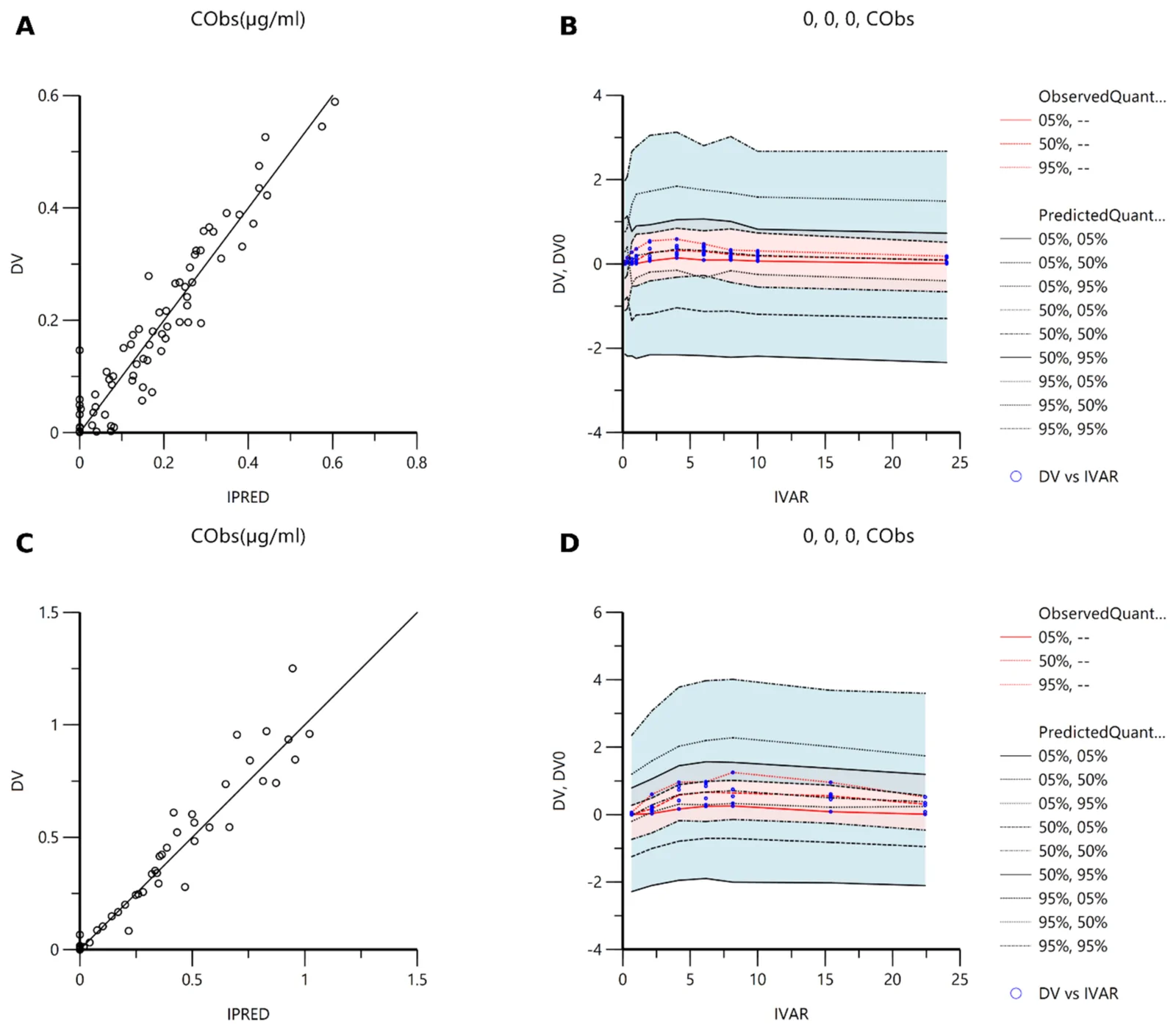
Goodness-of-fit diagnostics and visual predictive checks for the ciprofloxacin population pharmacokinetic models. (**A**) Observed versus individual predicted ciprofloxacin plasma concentrations. (**B**) Visual predictive check (VPC) for the ciprofloxacin plasma model. (**C**) Observed versus individual predicted ciprofloxacin interstitial fluid (ISF) concentrations. (**D**) Visual predictive check (VPC) for the ciprofloxacin ISF model. Open circles represent observed concentrations. In the VPC plots, the red lines indicate the observed 5th, 50th, and 95th percentiles, while the black lines and shaded areas represent the corresponding simulated percentiles and their 95% confidence intervals.

Visual predictive checks demonstrated that the observed concentration-time data were adequately described by the simulated median and percentile profiles. Observed median concentrations were generally contained within the simulated 95% confidence intervals throughout the sampling period. The simulated 5th–95th percentile intervals were relatively broad in several models, particularly for enrofloxacin in plasma and ISF, indicating substantial variability in the observed concentration data. Goodness-of-fit diagnostics based on observed versus individual predicted concentrations (DV vs. IPRED) and visual predictive checks (VPCs) for the final models are shown in Figure 3 and Figure 4. Overall, the diagnostic plots supported the adequacy of the final population pharmacokinetic models.

### 2.4. Probability of Target Attainment Analysis

Probability of target attainment decreased with increasing MIC values for both enrofloxacin and ciprofloxacin in plasma and ISF. For both analytes, PTA values were consistently higher in ISF than in plasma, resulting in higher MIC thresholds associated with adequate target attainment (PTA ≥90%).

The magnitude of target attainment depended on the PK/PD index and target threshold applied. For both compounds, lower AUC_0–24_/MIC targets (≥30 and ≥40) were achieved at higher MIC values than the more stringent AUC_0–24_/MIC targets (≥100 and ≥125). Differences were also observed between AUC_0–24_/MIC- and C_max_/MIC-based targets, indicating that the selected PK/PD index influenced the MIC threshold associated with adequate target attainment.

Enrofloxacin achieved the highest PTA values overall, particularly in ISF, where adequate target attainment was maintained across a broader MIC range than in plasma. Although PTA values for ciprofloxacin were lower than those for enrofloxacin, adequate target attainment was still achieved at low MIC values for several PK/PD targets. In ISF, ciprofloxacin achieved PTA ≥90% at MIC values up to 0.06 µg/mL for selected PK/PD targets, whereas lower MIC thresholds were observed in plasma.

Overall, PTA outcomes were influenced by both the biological matrix and the PK/PD target applied, with the highest target attainment observed for enrofloxacin in ISF and the lowest for ciprofloxacin in plasma (see Table 5 and Table 6).

## 3. Discussion

In the present study, the pharmacokinetics of enrofloxacin and its active metabolite ciprofloxacin were characterized in plasma and interstitial fluid following oral administration of enrofloxacin at a target dose of 20 mg/kg in dogs. The principal finding was that both enrofloxacin and ciprofloxacin achieved higher exposure in ISF than in plasma, accompanied by delayed peak concentrations in the tissue compartment. These pharmacokinetic differences translated into higher PTA estimates in ISF than in plasma across most evaluated PK/PD targets and MIC values. Together, these results suggest that extracellular tissue exposure, as represented by ISF concentrations, may differ substantially from plasma exposure and may therefore influence PK/PD target attainment assessments for fluoroquinolones. In addition, the study expands the available information on enrofloxacin disposition at a high oral dosage regimen and provides a framework for evaluating plasma and ISF PK/PD target attainment in dogs.

### 3.1. Plasma and Interstitial Fluid Exposure of Enrofloxacin

To place the present findings into context, comparison with the limited available canine literature reveals both expected and unexpected pharmacokinetic features. While the plasma disposition of enrofloxacin generally followed previously reported patterns, marked differences were observed with respect to extracellular exposure and the relationship between plasma and ISF concentrations.

The plasma pharmacokinetic profile of enrofloxacin was broadly consistent with previous canine investigations. Earlier studies by Küng et al. [12], Hauschild et al. [14], Sumano et al. [15], and Bidgood and Papich [11] demonstrated systemic absorption of enrofloxacin following oral administration and measurable formation of ciprofloxacin. As expected, systemic exposure exceeded that reported following conventional 5 mg/kg oral dosing regimens. Plasma peak concentrations reached 2.94 µg/mL, compared with 1.16 µg/mL reported by Küng et al., 1.24 µg/mL by Bidgood and Papich, and 1.89 µg/mL by Hauschild et al. Similarly, plasma AUC_0–24_ was 21.64 µg × h/mL, exceeding the values reported by Hauschild et al. (7.42 µg × h/mL), Bidgood and Papich (4.45 µg × h/mL), and Sumano et al. (8.02 µg × h/mL). These differences are consistent with the four-fold higher dose administered in the present study and indicate substantially greater systemic exposure while maintaining the overall pharmacokinetic characteristics previously described for enrofloxacin in dogs.

The elimination half-life observed in the present study (5.89 h) was longer than those reported by Küng et al. (2.40 h) [12], Bidgood and Papich (2.23 h) [11], Hauschild et al. (3.18 h) [14], and Sumano et al. (2.40 h) [15], but was comparable to the values reported by Boothe et al. following administration of 10 and 20 mg/kg enrofloxacin (5.05 and 4.65 h, respectively) [13]. Because non-compartmental half-life estimates are highly dependent on characterization of the terminal elimination phase, differences in sampling schedules, terminal-phase data selection, and analytical sensitivity may contribute to inter-study variability [21]. Interestingly, the prolonged plasma half-life observed in the present study coincided with prolonged persistence in ISF, suggesting that tissue distribution kinetics may contribute to the overall disposition profile observed following high-dose administration. Although measurable enrofloxacin concentrations remained at the final 24 h sampling time, the extrapolated fraction of AUC_inf_ remained low (mean <13% for all analytes and matrices), indicating that the AUC_inf_ estimates were largely supported by observed concentration-time data. Nevertheless, λ_z_, terminal half-life, and AUC_inf_ remain inherently more sensitive to terminal phase characterization than AUC_0–24_ and should therefore be interpreted with appropriate caution.

An exception among the available studies was the report of Boothe et al. [13], who observed substantially higher plasma exposure following administration of a target dose of approximately 20 mg/kg enrofloxacin (C_max_ 4.74 µg/mL; AUC_0–24_ 35.83 µg × h/mL) than that observed in the present study (2.94 µg/mL and 21.64 µg × h/mL, respectively). Similar differences were reported for ciprofloxacin. As these values also exceeded exposure estimates reported in several other canine studies, the discrepancy most likely reflects substantial inter-study variability rather than a deviation unique to the present investigation. Differences in study populations may have contributed to this variability, as Boothe et al. used Greyhounds, whereas most other available canine studies included Beagles or mixed-breed dogs.

To our knowledge, only two previous canine ultrafiltration studies have evaluated enrofloxacin concentrations in ISF, both following administration of 5 mg/kg enrofloxacin [11,14]. Consequently, the higher extracellular concentrations observed in the present study were expected. Enrofloxacin ISF peak concentrations reached 3.77 µg/mL, compared with 0.70 µg/mL reported by Bidgood and Papich [11] and 0.59 µg/mL reported by Hauschild et al. [14]. Likewise, ISF exposure (AUC_0–24_) reached 44.93 µg × h/mL, compared with 5.16 µg × h/mL and 5.19 µg × h/mL in the studies of Bidgood and Papich and Hauschild et al., respectively. More informative than these quantitative differences, however, was the remarkable consistency in distribution kinetics. All available ultrafiltration studies demonstrated delayed equilibration between plasma and ISF, reflected by later T_max_ values in the extracellular compartment. In the present study, T_max_ increased from 2.88 h in plasma to 4.49 h in ISF, compared with 0.94 h to 4.40 h in the study of Bidgood and Papich and from 1.66 h to 5.33 h in the study of Hauschild et al. Together, these observations support the view that delayed distribution into ISF represents a characteristic feature of enrofloxacin disposition in dogs.

Although all available studies demonstrated delayed distribution into the extracellular compartment, the relationship between plasma and ISF exposure differed substantially among investigations. Hauschild et al. reported lower enrofloxacin exposure in ISF than in plasma (AUC_ISF_/AUC_plasma_ 0.70), whereas Bidgood and Papich reported slightly higher ISF exposure (1.16). In the present study, analysis of the six dogs with paired plasma and ISF observations yielded a mean AUC_ISF_/AUC_plasma_ ratio of 1.87. Because this ratio was calculated exclusively from paired observations, it is not identical to the ratio obtained from the overall group means presented in Table 1. These findings suggest that extracellular exposure may vary considerably among studies and indicate that plasma concentrations do not necessarily provide a reliable surrogate for target-site drug exposure [16,22]. From a pharmacodynamic perspective, this observation is particularly relevant because antibacterial efficacy is ultimately determined by drug concentrations at the site of infection rather than in the central compartment [16].

Together with the higher ISF concentrations observed during the terminal phase, the paired-data analysis supports greater overall extracellular exposure following high-dose oral administration. However, this finding warrants careful interpretation. Concentrations obtained by in vivo ultrafiltration are generally accepted to represent protein-unbound drug concentrations within the extracellular fluid compartment [11,14], whereas the plasma concentrations measured in the present study represent total drug concentrations. Consequently, plasma and ISF exposures were not compared on a strictly equivalent basis. In addition, the present study evaluated concentration-time profiles following a single oral dose rather than under steady-state conditions, where instantaneous equilibrium between plasma and interstitial fluid cannot be assumed. Consistent with this interpretation, both enrofloxacin and ciprofloxacin exhibited delayed T_max_ values together with more prolonged concentration-time profiles in ISF, indicating slower equilibration and persistence within the extracellular compartment. These pharmacokinetic characteristics provide a plausible explanation for the greater ISF AUC observed following high-dose oral administration. Nevertheless, because plasma protein binding was not determined experimentally, direct comparison between free plasma and ISF exposure was not possible, and the relative contributions of plasma protein binding and tissue pharmacokinetics to the observed ISF-to-plasma exposure ratio cannot be fully resolved. Correction of plasma concentrations using published protein binding values was intentionally not performed because protein binding may vary between studies as a consequence of methodological and experimental differences. Consequently, comparison of measured total plasma concentrations with directly measured ultrafiltrate concentrations was considered the most transparent approach, although this requires cautious interpretation of plasma–ISF differences. Nevertheless, direct determination of plasma protein binding and free plasma concentrations would have allowed a more rigorous comparison between systemic and target-site exposure and should be considered in future investigations.

A further methodological consideration concerns the absence of analyte-specific validation of membrane passage and adsorption for the ultrafiltration probes. In contrast to microdialysis, in vivo ultrafiltration collects extracellular fluid without dilution by a continuously perfused solution; therefore, the conventional relative-recovery correction used for microdialysis was not applied. This approach is consistent with previous canine studies using the same ultrafiltration methodology, in which ISF samples were analyzed directly and no in vivo probe-recovery correction was reported [11,14]. Bidgood and Papich reported analytical recovery for their HPLC assay and used an ultrafiltration device in vitro to determine plasma protein binding, but did not describe an analyte-specific correction of the in vivo ISF concentrations for probe recovery [11]. Similarly, Hauschild et al. analyzed undiluted ISF samples using matrix-matched calibration without reporting an in vivo probe-recovery correction [14]. Nevertheless, because specific membrane adsorption and passage experiments were not performed for enrofloxacin and ciprofloxacin in the present study, potential analyte loss within the probe or connecting tubing cannot be completely excluded. The absolute ISF concentrations, ISF-to-plasma exposure ratios, and ISF-based PTA estimates should therefore be interpreted with this limitation in mind, and analyte-specific membrane and adsorption testing should be included in future investigations.

The present pharmacokinetic data were obtained in healthy dogs and therefore describe drug distribution under physiological conditions. In contrast, bacterial skin infections are characterized by inflammatory changes that may alter local tissue perfusion, capillary permeability, extracellular fluid composition, and consequently antimicrobial penetration into the site of infection. Moreover, infection and inflammation have been shown to modify systemic drug disposition through effects on drug-metabolizing enzymes, transporters, and other physiological processes, although the magnitude and direction of these changes depend on the disease process and affected tissue [23]. Likewise, interpretation of antimicrobial concentrations measured in interstitial fluid should consider the pathophysiological characteristics of infected tissues, since tissue penetration observed under physiological conditions may not fully reflect drug exposure at sites of active infection. Therefore, the ISF concentration-time profiles reported here should be regarded as a physiological reference, and confirmation of these findings in dogs with naturally occurring bacterial skin infections would be valuable before direct clinical extrapolation [23,24].

Finally, the present study was designed to characterize the pharmacokinetics of high-dose enrofloxacin rather than to evaluate its safety or tolerability. Although no clinically apparent adverse events were observed in the remaining animals following exclusion of one dog that vomited shortly after dosing, these observations should not be interpreted as a formal safety assessment. Importantly, the administered dose corresponds to the upper limit of the FDA-approved dosage range for dogs, although it exceeds the currently authorized oral dose in the European Union. Therefore, the safety observations reported here should be regarded solely as incidental findings within a pharmacokinetic study rather than evidence supporting the overall tolerability of the regimen.

### 3.2. Plasma and Interstitial Fluid Exposure of Ciprofloxacin

Plasma ciprofloxacin exposure generally followed the expected pattern of metabolite formation after enrofloxacin administration, although the increase in exposure was modest relative to the four-fold higher enrofloxacin dose administered in the present study. Following oral administration of a target dose of approximately 20 mg/kg enrofloxacin, plasma ciprofloxacin C_max_ and AUC_0–24_ reached 0.37 µg/mL and 4.35 µg × h/mL, respectively. These values were slightly higher than those reported by Bidgood and Papich [11] after administration of 5 mg/kg enrofloxacin (C_max_ 0.36 µg/mL and AUC_0–24_ 2.61 µg × h/mL, respectively) and exceeded those reported by Küng et al. [12] (C_max_ 0.29 µg/mL; AUC_inf_ 2.27 µg × h/mL). In contrast, substantially greater ciprofloxacin exposure was reported by Boothe et al. [13], with C_max_ values ranging from 1.35 to 2.00 µg/mL and AUC_0–24_ values ranging from 15.30 to 35.22 µg × h/mL across the investigated dose range. As similarly elevated enrofloxacin exposures were reported in the same study, these findings are consistent with the broader between-study differences observed for the parent compound. Overall, the present findings indicate that ciprofloxacin exposure increased following high-dose enrofloxacin administration, but to a considerably lesser extent than exposure to the parent compound.

A similar pattern was observed in ISF. Ciprofloxacin ISF peak concentrations reached 0.77 µg/mL in the present study, compared with 0.35 µg/mL reported by Bidgood and Papich [11], whereas ISF AUC_0–24_ was 10.68 µg × h/mL compared with 3.89 µg × h/mL reported previously. As observed for enrofloxacin, ciprofloxacin distribution into ISF was delayed, with T_max_ increasing from 4.50 h in plasma to 8.91 h in ISF in the present study, compared with 3.00 h and 6.80 h, respectively, in the study of Bidgood and Papich. Despite the lower relative contribution of ciprofloxacin, these findings indicate sustained extracellular exposure and suggest that distribution into ISF broadly paralleled that observed for enrofloxacin.

The lower relative contribution of ciprofloxacin was reflected in the AUC_CIP_/AUC_ENR_ ratio. In the present study, this ratio was 20.1% in plasma and 23.8% in ISF, whereas previously reported values were approximately 58% in the studies of Küng et al. [12] and Bidgood and Papich [11] and nearly 100–135% in the study of Boothe et al. [13]. Importantly, this finding should not be interpreted as direct evidence of reduced ciprofloxacin formation, because metabolite exposure reflects both formation and subsequent disposition processes [25]. Currently available canine pharmacokinetic data also do not support a straightforward dose-dependent limitation of enrofloxacin-to-ciprofloxacin conversion, as ciprofloxacin exposure increased rather than plateaued across the investigated dose range. Consequently, the comparatively low AUC_CIP_/AUC_ENR_ ratio observed in the present study should be interpreted as a pharmacokinetic observation rather than as evidence of a specific metabolic mechanism [26].

Overall, ciprofloxacin contributed a smaller proportion of total fluoroquinolone exposure than reported in earlier canine studies, whereas enrofloxacin remained the predominant contributor in both plasma and ISF. Nevertheless, measurable and sustained ciprofloxacin concentrations were present in both matrices, providing relevant context for interpretation of the subsequent population pharmacokinetic modeling and PTA analyses.

### 3.3. Nonlinear Mixed-Effects Pharmacokinetic Modeling and Monte Carlo Simulations

Nonlinear mixed-effects pharmacokinetic modeling provided a consistent description of enrofloxacin and ciprofloxacin concentration-time profiles in both plasma and interstitial fluid and generated exposure distributions that subsequently enabled population-based PK/PD evaluation. The population models should be regarded as exploratory because they were developed from a limited experimental dataset and were primarily intended to generate exposure distributions for simulation-based PTA analysis rather than to establish definitive population pharmacokinetic models for dogs. Although separate empirical models were developed for each analyte-matrix combination, these models were intended solely to describe the observed concentration-time profiles within each dataset and should not be interpreted as independent physiological representations of plasma-to-interstitial fluid distribution or parent-to-metabolite conversion. A common modeling strategy adequately described all datasets. More complex structural models were explored during model development but did not result in sufficient improvement of model performance to justify the inclusion of additional parameters. Consequently, one-compartment models with first-order absorption and elimination were retained for all final analyses. Diagnostic plots and visual predictive checks demonstrated good agreement between observed and simulated concentrations, indicating that the final models adequately captured both the central tendency and the variability of the observed pharmacokinetic profiles [27,28]. Importantly, the structural characteristics identified by the population models were consistent with the findings of the non-compartmental analyses. The lower empirical K_a_ estimates and longer lag-time parameters in the ISF models were consistent with the later appearance and delayed peak concentrations observed in ISF. However, because the plasma and ISF datasets were modeled separately, these parameters should not be interpreted as mechanistic estimates of plasma-to-tissue distribution. Furthermore, the predominantly low shrinkage values observed for the principal random effects supported the reliability of individual parameter estimates despite the substantial interindividual variability retained in the final models.

One aspect of the ciprofloxacin population pharmacokinetic model, however, requires careful interpretation. Because ciprofloxacin was formed in vivo from enrofloxacin rather than administered as a known dose, the apparent clearance (CL/F) and apparent volume of distribution (V/F) estimated by the model are intrinsically confounded by the unknown fraction of enrofloxacin converted to ciprofloxacin (formation fraction, fm). Consequently, these parameters should not be interpreted as intrinsic physiological disposition parameters but rather as empirical descriptors of the observed metabolite concentration-time profiles. Nevertheless, the model adequately described the observed data and was therefore suitable for simulation-based PTA analyses.

Considerable interindividual variability was already evident in the observed concentration-time profiles and in the non-compartmental pharmacokinetic analysis, as reflected by the relatively large standard deviations of several exposure parameters. Consequently, the NCA-derived pharmacokinetic parameters should be interpreted primarily as descriptive summaries of the study population rather than precise estimates applicable to individual animals. This variability provided the rationale for the subsequent population pharmacokinetic modeling, which explicitly quantified between-subject variability and served as the basis for the Monte Carlo simulations.

One of the most notable findings of the population analyses was the substantial interindividual variability associated with several pharmacokinetic parameters, particularly apparent clearance. Importantly, this observation was fully consistent with the non-compartmental analyses, which already demonstrated considerable variability in systemic and tissue exposure. Thus, the population models did not introduce variability that was absent from the original dataset but rather quantified heterogeneity already present in the observed pharmacokinetic data. Similar findings have been reported in canine fluoroquinolone studies, including population pharmacokinetic analyses of marbofloxacin and investigations of oral ciprofloxacin, where variability in systemic exposure substantially influenced PK/PD target attainment [29,30]. The observed variability likely reflected both biological variability and the use of a fixed tablet strength. Because all dogs received the same oral dose (225 mg), the administered dose ranged from 15.3 to 21.0 mg/kg depending on individual body weight. Consequently, part of the observed interindividual variability in systemic exposure may have originated from dose differences rather than biological pharmacokinetic variability alone. To investigate this possibility, body weight was formally evaluated as a continuous covariate on apparent clearance (CL/F) and apparent volume of distribution (V/F) during population pharmacokinetic model development. Although inclusion of body weight resulted in minor numerical improvements in selected analyte-matrix models, these effects were not consistent across analytes and matrices and were not uniformly supported by improvements in model diagnostics or numerical stability. Therefore, body weight was not retained in the final parsimonious models. Nevertheless, because the Monte Carlo simulations propagate the variability estimated by the final population pharmacokinetic models, variability associated with fixed-dose administration is likely reflected in the simulated exposure distributions and, consequently, in the calculated PTA values. Importantly, the PTA analysis was designed to reflect the variability expected under the dosing regimen applied in the present study rather than an idealized weight-adjusted dosing strategy. Although weight-adjusted dosing could potentially reduce exposure variability, confirmation of this hypothesis would require studies including a larger number of dogs and a broader body-weight range. Similar observations have been reported in other veterinary population pharmacokinetic investigations, where biologically plausible covariates reduced, but did not eliminate, interindividual variability [31,32].

Beyond characterization of variability, an important strength of the present modeling approach was the generation of population-based exposure distributions for both plasma and ISF. While most veterinary PK/PD investigations rely exclusively on plasma pharmacokinetics, inclusion of ISF data enabled subsequent evaluation of target attainment based on tissue exposure rather than systemic exposure alone. This distinction is particularly relevant for antimicrobial agents because interstitial fluid represents the effect site for antibiotics targeting extracellular pathogens, and only free drug concentrations at the site of infection are directly linked to antibacterial activity [16,33]. Previous studies investigating fluoroquinolone disposition in dogs have similarly emphasized the importance of ISF concentrations as a pharmacologically relevant surrogate for target-site exposure and antimicrobial efficacy [14]. Consequently, the availability of separate plasma- and ISF-based exposure distributions provided a unique opportunity to compare PTA predictions derived from systemic and tissue pharmacokinetics within the same study framework.

Incorporation of the observed variability into Monte Carlo simulations enabled assessment of PK/PD target attainment across the entire exposure distribution represented within the study population rather than on the basis of mean pharmacokinetic parameters alone. Importantly, the simulations were based on exposure distributions derived directly from the final population models and therefore reflected the variability observed in the study animals rather than externally assumed distributions. Because the population pharmacokinetic models were developed from a relatively small number of animals, particularly for the ISF dataset, the estimated interindividual variability and resulting exposure distributions should be interpreted with appropriate caution. Consequently, the Monte Carlo simulations should be regarded as exploratory and representative of the variability observed within the present dataset rather than definitive predictions for the broader canine population. Larger studies will be required to confirm parameter stability, model robustness, and the generalizability of these simulation-based PTA estimates. Consequently, although the PTA simulations explicitly incorporated the observed pharmacokinetic variability, the predicted probabilities should be regarded as exploratory and representative of the variability captured within the present dataset rather than definitive estimates for the wider canine population. Similar simulation-based approaches have been successfully applied in veterinary fluoroquinolone investigations to evaluate the impact of pharmacokinetic variability on antimicrobial target attainment [10,31,34]. In the present study, the consequences of exposure variability became evident in the subsequent PTA analyses, where differences in exposure translated directly into differences in predicted target attainment. Despite the relatively small study population, the low η-shrinkage values and satisfactory diagnostic performance suggest that the models adequately described the observed data.

Nevertheless, an important limitation of the present simulations should be acknowledged. The present population pharmacokinetic models and Monte Carlo simulations were based exclusively on concentration-time data collected during the first 24 h following a single oral administration. Consequently, the reported PTA estimates describe target attainment during the initial dosing interval and should not be directly extrapolated to repeated q24h administration or steady-state conditions. Although repeated-dose simulations could be generated mathematically, such predictions would rely on assumptions of time-invariant pharmacokinetics and could not be evaluated against experimental repeated-dose observations. This uncertainty is further increased by the relatively limited number of animals included in the present study, particularly for the ISF dataset. Therefore, we considered steady-state simulations insufficiently supported by the available experimental data and restricted the analyses to the experimentally observed single-dose setting. Future studies incorporating repeated-dose pharmacokinetics, steady-state sampling, and clinical outcome data will be required to establish the applicability of the present findings to multi-day antimicrobial therapy.

### 3.4. PK/PD Target Attainment in Plasma and Interstitial Fluid

The PTA analysis demonstrated that interpretation of PK/PD target attainment was strongly influenced by both the biological matrix and the PK/PD endpoint applied. Although high probabilities of target attainment were observed at low MIC values across all evaluated targets, the MIC threshold associated with ≥90% PTA varied substantially depending on the selected PK/PD index. This finding is consistent with previous investigations demonstrating that application of different fluoroquinolone efficacy targets may lead to markedly different conclusions regarding the susceptibility range expected to be treatable [35,36]. As expected, the less stringent AUC_0–24_/MIC targets (≥30 and ≥40) produced higher PTA values and extended the MIC range associated with adequate target attainment compared with the more conservative AUC_0–24_/MIC ≥100 and ≥125 targets. Differences were also observed between C_max_/MIC- and AUC_0–24_/MIC-based assessments, emphasizing the importance of endpoint selection when interpreting PK/PD target attainment. Although both indices have historically been used for fluoroquinolones, contemporary PK/PD analyses generally place greater emphasis on AUC/MIC because it integrates overall antimicrobial exposure and has been more consistently associated with antibacterial efficacy and suppression of resistance emergence [19,37].

The most important finding of the present study was the consistently higher PTA observed in ISF than in plasma. This comparison should be interpreted with appropriate caution because plasma-based PTA was derived from total plasma concentrations, whereas ISF-based PTA was derived from ultrafiltrate concentrations that are generally considered to represent unbound extracellular drug. For enrofloxacin, ISF-based PTA exceeded plasma-based PTA across all evaluated PK/PD targets, with the largest differences observed for the more conservative AUC/MIC endpoints. Depending on the selected target, MIC thresholds associated with ≥90% PTA were approximately two- to four-fold higher in ISF than in plasma. These findings indicate that target-site exposure was consistently more favorable than would have been predicted from plasma pharmacokinetics alone. Because ISF concentrations obtained by ultrafiltration closely represent unbound extracellular drug concentrations, which are directly relevant for antimicrobial activity against extracellular pathogens, the present data suggest that plasma-based PK/PD assessments may underestimate pharmacologically relevant exposure at the site of infection [16,33]. Importantly, the present study assessed drug concentrations directly in ISF following administration of high-dose enrofloxacin, providing information from a biologically relevant compartment that is rarely incorporated into veterinary PTA analyses.

Comparison of the PTA results with contemporary susceptibility breakpoint assessments further emphasizes the clinical relevance of these observations. Recent PK/PD-based reevaluation of canine fluoroquinolone breakpoints suggested susceptible MIC thresholds of approximately 0.06 µg/mL for conventional 5 mg/kg enrofloxacin dosing and up to 0.25 µg/mL for 20 mg/kg dosing when PTA-based approaches were applied [20]. The present findings generally support the conclusion that high-dose enrofloxacin substantially expands the MIC range associated with adequate target attainment. Notably, ISF-based PTA achieved ≥90% target attainment at MIC values up to 0.125 µg/mL even when conservative AUC/MIC targets were applied, whereas plasma-based analyses suggested considerably lower thresholds. Although the present dataset is insufficient to support formal revision of susceptibility breakpoints, these results provide independent evidence that target-site exposure may support PK/PD target attainment across a broader MIC range than would be inferred from plasma concentrations alone. Consequently, susceptibility assessments based exclusively on plasma pharmacokinetics may represent a conservative estimate of antibacterial exposure at the site of infection.

Although ciprofloxacin consistently produced lower PTA values than enrofloxacin, this observation was expected given its role as a metabolite rather than the primary administered compound. Nevertheless, adequate target attainment was still achieved at lower MIC values, particularly in ISF, indicating that ciprofloxacin may contribute to the overall antibacterial activity following enrofloxacin administration despite accounting for a smaller proportion of total fluoroquinolone exposure than the parent compound [13,20].

The PTA analyses for enrofloxacin and ciprofloxacin were intentionally performed separately. Previous PK/PD investigations have combined parent-drug and metabolite exposure by summing their concentrations or AUC values when comparable antibacterial potency or identical MIC values could be assumed, and combined exposure has also been considered in canine fluoroquinolone breakpoint analyses [20]. A potency-adjusted approach could alternatively be expressed by weighting ciprofloxacin exposure according to the ratio of the enrofloxacin and ciprofloxacin MICs for the relevant pathogen. However, such an analysis requires organism-specific, paired susceptibility data and assumes that the antibacterial contributions of the two compounds are additive. Because the present study was not directed at a single bacterial species and did not include paired enrofloxacin and ciprofloxacin MIC distributions, no scientifically justified, generally applicable potency-weighting factor could be defined. Consequently, the compound-specific PTA estimates presented here may not fully capture the total fluoroquinolone activity resulting from the combined exposure to enrofloxacin and its active metabolite and should therefore be interpreted as analyte-specific rather than overall fluoroquinolone target attainment.

The present findings should nevertheless be interpreted in light of the relatively small number of animals included in the study. In particular, paired plasma-ISF comparisons were based on only six animals, resulting in limited statistical resolution of the Wilcoxon signed-rank test. Under these conditions, the minimum attainable two-sided *p*-value is 0.03125; consequently, statistically significant findings primarily indicate consistent within-animal directional differences across all paired observations rather than providing finely graded statistical evidence. Although the observed PTA patterns were internally consistent and biologically plausible, the available dataset remains insufficient to support formal modification of currently accepted susceptibility breakpoints [38]. Consequently, the ISF-based PTA results should be considered hypothesis-generating and warrant confirmation in larger pharmacokinetic datasets specifically designed for breakpoint evaluation. Collectively, the data indicate that high-dose enrofloxacin achieves favorable PK/PD target attainment across a broader MIC range than conventional dosage regimens and highlight the value of incorporating target-site exposure into future PK/PD-based optimization of fluoroquinolone dosing strategies and susceptibility breakpoint assessment in dogs.

## 4. Materials and Methods

### 4.1. Animals

Nine purpose-bred, healthy adult Beagle dogs were included in the study. All dogs were intact males with body weights of 11.7 ± 1.5 kg (range: 9.3–14.7 kg). Prior to enrollment, all animals underwent veterinary examination and were determined to be clinically healthy. Dogs were housed at the Aurigon Labs Ltd. facility (Dunakeszi, Hungary) throughout the study period. Animals were acclimatized to the experimental conditions for at least 7 days before study initiation. During the acclimatization period, dogs were pair-housed, whereas during dosing and pharmacokinetic sampling they were housed individually while maintaining visual, olfactory, and auditory contact with conspecifics. Dogs received a commercial dry diet (Ecopet Natural, Farmina Pet Foods, Budapest, Hungary) once daily; on treatment days, animals were fasted for at least 12 h before dosing and food was offered approximately 3 h after drug administration. Tap water was available ad libitum throughout the study. Environmental enrichment was provided using resting boxes and toys. Animal rooms were maintained under a 12 h light/12 h dark cycle at 20 ± 5 °C and 30–70% relative humidity. The study protocol was reviewed and approved by the Local Animal Welfare Committee of the University of Veterinary Medicine Budapest and by the Government Office of Pest County, Food Chain Safety, Plant Protection and Soil Conservation Directorate, Budapest, Hungary on 17 July 2024 (approval number: PE/EA/00603-4/2024). No formal a priori sample size calculation was performed. The sample size was selected based on comparable exploratory pharmacokinetic studies reported in the veterinary literature and was considered sufficient to characterize the pharmacokinetic profiles of enrofloxacin and ciprofloxacin following a single oral administration.

### 4.2. Study Design

The study was designed as a single-dose pharmacokinetic investigation. Following a 12 h fasting period, dogs received a single oral dose of enrofloxacin as Baytril Flavour 150 mg tablets (KVP Pharma + Veterinär Produkte GmbH, Kiel, Germany). The target dose was 20 mg/kg; however, due to tablet size limitations, all dogs received a fixed dose of 225 mg (1.5 tablets). Immediately after tablet administration, 10 mL of water was administered orally to ensure complete ingestion of the dose. Food was withheld for an additional 3 h after drug administration, while water remained available ad libitum throughout the study. Plasma and ISF samples were collected over a 24 h period following enrofloxacin administration. Animals were monitored throughout the study by trained veterinary personnel for their general health and well-being. No predefined humane endpoints were established because this was a single-dose pharmacokinetic study involving healthy purpose-bred research dogs. Following completion of the study, all animals recovered uneventfully and were returned to routine housing at the research facility.

### 4.3. Catheter and Ultrafiltration Probe Placement

Approximately 18–24 h before enrofloxacin administration, dogs were anesthetized for placement of a central venous catheter (CVC) and an ultrafiltration probe (UFP). Anesthesia was induced by intravenous administration of butorphanol (Nalgosed 10 mg/mL; Bioveta, Ivanovice na Hané, Czech Republic) at 0.3 mg/kg and propofol (1% MCT/LCT Fresenius; Fresenius Kabi, Bad Homburg, Germany) at 5 mg/kg. A central venous catheter was inserted into the external jugular vein for serial blood collection, while an ultrafiltration probe (UF-3-12; BASi Research Products, West Lafayette, IN, USA) was implanted subcutaneously in the interscapular region for collection of interstitial fluid samples. Following placement, both devices were secured and remained in place throughout the 24 h pharmacokinetic sampling period. After completion of sample collection, the catheter and ultrafiltration probe were removed.

### 4.4. Plasma Sampling

Blood samples (approximately 2 mL) were collected via the central venous catheter into lithium-heparin blood collection tubes (Vacuette Li-Heparin; Greiner Bio-One, Kremsmünster, Austria) at 0 (pre-dose), 10, 20, and 40 min, as well as 1, 2, 4, 6, 8, 10, and 24 h after enrofloxacin administration. Following collection, samples were gently mixed with the anticoagulant, maintained on ice-cold water, and centrifuged within 60 min at 2000× *g* for 20 min at 4 °C. Plasma was separated and transferred into polypropylene tubes and stored below −70 °C until analysis.

### 4.5. Interstitial Fluid Sampling

ISF samples were collected using an in vivo ultrafiltration probe (UF-3-12; BASi Research Products, West Lafayette, IN, USA). The probe was implanted subcutaneously in the interscapular region approximately 18–24 h before enrofloxacin administration, as described above. The ultrafiltration probe consisted of a semi-permeable membrane allowing collection of protein-free ISF by application of negative pressure through an evacuated collection tube. ISF samples were collected into lithium-heparinized collection tubes (Vacuette Li-Heparin; Greiner Bio-One, Kremsmünster, Austria) at 0 (pre-dose), 1, 2, 4, 6, 8, 10, 22.5, and 24 h after drug administration by replacing the evacuated collection tube at each sampling interval. Following collection, samples were gently mixed with the anticoagulant, maintained on ice-cold water, and centrifuged within 60 min at 2000× *g* for 20 min at 4 °C. The ISF samples were then transferred into polypropylene tubes and stored below −70 °C until analysis. Ultrafiltrate samples were analyzed as collected without applying probe recovery correction. Unlike microdialysis, in vivo ultrafiltration collects protein-free extracellular fluid directly and therefore does not require correction for dilution by a perfusion solution. This approach is consistent with previous canine ultrafiltration studies by Bidgood and Papich [11] and Hauschild et al. [14], in which ultrafiltrate concentrations were analyzed without recovery correction. No analyte-specific in vitro adsorption or membrane recovery experiments were performed for enrofloxacin or ciprofloxacin.

### 4.6. LC-MS/MS Analysis

Quantitative analyses were performed using a Sciex 6500 QTRAP tandem mass spectrometer (SCIEX, Framingham, MA, USA) coupled to an Agilent 1100 HPLC system (Agilent Technologies, Santa Clara, CA, USA). Measurements were carried out under positive electrospray ionization (ESI+) conditions in multiple reaction monitoring (MRM) mode. Source parameters were as follows: nebulizer gas (GS1), drying gas (GS2), and curtain gas (CUR) were set to 40, 40, and 45 arbitrary units, respectively. The ion spray voltage was 5500 V and the source temperature was 450 °C. The monitored MRM transitions (Q1/Q3) were 332.1/245.1 and 332.1/288.0 for ciprofloxacin, and 360.09/316.0 and 360.09/244.9 for enrofloxacin. Collision energies for the respective transitions were 33, 35, 27, and 27 eV. The dwell time was 80 ms for each transition. Chromatographic separation was achieved on an Agilent XDB-C18 column (75 × 4.6 mm, 5 µm; Agilent Technologies, Santa Clara, CA, USA) using water (eluent A) and acetonitrile (eluent B), both containing 0.1% formic acid. The initial mobile phase composition was 90:10 (A:B). The proportion of eluent B was increased to 95% over 3 min and maintained for 1 min. The mobile phase was then returned to the initial composition over 0.2 min and equilibrated for 2.8 min before the next injection. The flow rate was 0.7 mL/min. Proteins were precipitated by adding three volumes of methanol to the samples. After vortex mixing, the samples were centrifuged at 13,000 rpm for 5 min, and the resulting supernatants were transferred to autosampler vials. An aliquot of 5 µL was injected for analysis. Instrument control and data acquisition were performed using Analyst software (Analyst 1.6.3, AB SCIEX, Concord, ON, Canada), while data processing was carried out using MultiQuant software (MultiQuant 2.1, AB SCIEX, Concord, ON, Canada).

### 4.7. Bioanalytical Method Validation

A fit-for-purpose partial validation of the LC–MS/MS method was performed for the determination of enrofloxacin and ciprofloxacin in canine plasma and interstitial fluid (ISF). Validation experiments were conducted according to the relevant principles of the ICH M10 guideline and included assessment of selectivity, calibration-curve performance and quantitation limits, accuracy and precision, matrix effects, carryover, and stability. Quantification was performed without an internal standard. A detailed description of the validation procedure and the corresponding results is provided in the Supplementary Materials (Validation Method, Supplementary Tables S1 and S2). The absence of an internal standard is acknowledged as a limitation of the analytical method and is considered in the interpretation of the study results.

### 4.8. Pharmacokinetic Analysis

Non-compartmental pharmacokinetic analysis (NCA) was performed using Phoenix WinNonlin version 8.5.2.4 (Certara USA, Princeton, NJ, USA). Plasma and ISF concentration-time data for enrofloxacin and ciprofloxacin were analyzed using an extravascular model and the linear-up/log-down trapezoidal method. Concentrations below the limit of quantification (BLQ) were treated as missing values.

Pharmacokinetic parameters including maximum concentration (C_max_), time to maximum concentration (T_max_), area under the concentration-time curve from 0 to 24 h (AUC_0–24_), area under the concentration-time curve extrapolated to infinity (AUC_inf_), area under the first moment curve extrapolated to infinity (AUMC_inf_), terminal elimination rate constant (λ_z_), terminal elimination half-life (t_1/2_), and mean residence time extrapolated to infinity (MRT_inf_) were calculated. The terminal elimination rate constant was estimated by log-linear regression of the terminal concentration-time data in Phoenix WinNonlin version 8.5.2.4 (Certara USA, Princeton, NJ, USA). At least three terminal data points were included in the estimation, and the selected terminal phase was verified by visual inspection. The percentage of AUC extrapolated beyond the last quantifiable concentration (%AUC_extrap_) was also recorded to assess the reliability of λ_z_ and AUC_inf_ estimates.

Because ISF samples represented predefined collection intervals, each ISF concentration was assigned to the midpoint (t_mid_) of the corresponding collection interval (t_corr_). To account for the dead volume of the ultrafiltration system, assigned sampling times were corrected according to

$$t_{corr}=t_{mid}-t_{UF}$$

where

$$t_{UF}=\frac{V_{dead}}{Q_{ISF}}$$

where V_dead_ is the dead volume of the ultrafiltration probe (70 µL) and Q_ISF_ is the mean ISF flow rate. Samples for which lag-time correction resulted in negative assigned times were excluded from post-dose pharmacokinetic analysis.

### 4.9. Population Pharmacokinetic Modeling

Exploratory nonlinear mixed-effects (NLME) pharmacokinetic modeling was performed using the NLME module of Phoenix WinNonlin version 8.5.2.4 (Certara USA, Princeton, NJ, USA). Separate models were developed for enrofloxacin and ciprofloxacin in plasma and ISF. Plasma models were based on data from eight dogs, whereas ISF models were based on data from six dogs.

For each analyte and matrix, one-compartment structural models with first-order absorption, first-order elimination, and absorption lag time were evaluated. Model parameters included the first-order absorption rate constant (K_a_), apparent volume of distribution (V/F), apparent clearance (CL/F), and absorption lag time (T_lag_).

Interindividual variability (IIV) was modeled using exponential random-effects models and was included on V/F, CL/F, and T_lag_ for enrofloxacin in plasma, on V/F and CL/F for enrofloxacin in ISF, on V/F and CL/F for ciprofloxacin in plasma, and on CL/F and K_a_ for ciprofloxacin in ISF. The magnitude of interindividual variability was expressed as the coefficient of variation (%CV) and calculated from the estimated variance (ω^2^) according to

$$\%CV=\sqrt{e^{\omega^{2}}-1}\times 100$$

A combined additive and multiplicative residual error model was used for enrofloxacin in plasma and ISF and for ciprofloxacin in ISF. An additive residual error model was used for ciprofloxacin in plasma. Models were estimated using the first-order conditional estimation extended least squares (FOCE ELS) algorithm. Approximate 95% confidence intervals for parameter estimates were derived from the Hessian-based variance-covariance matrix assuming asymptotic normality. Consequently, for parameters constrained to positive values (e.g., absorption rate constants), the lower confidence limits may extend below zero because the asymptotic approximation does not account for parameter boundary constraints.

Because all dogs received the same oral dose of 225 mg, differences in body weight resulted in variable administered doses when expressed on a body-weight basis (mg/kg). Therefore, body weight was formally evaluated as a continuous covariate on apparent clearance (CL/F) and apparent volume of distribution (V/F) using a median-centered power model. Covariate models were compared with the corresponding base models using changes in objective function value, Akaike information criterion (AIC), parameter precision, model stability, and reduction in unexplained interindividual variability. Only covariates providing a consistent and robust improvement were considered for inclusion in the final models.

The primary objective of model development was to obtain parsimonious empirical models suitable for Monte Carlo simulation and PTA analysis rather than mechanistic characterization of plasma-to-interstitial fluid distribution or enrofloxacin-to-ciprofloxacin conversion.

### 4.10. Model Evaluation

Model performance was evaluated using goodness-of-fit diagnostics and visual predictive checks (VPCs) generated in the NLME module of Phoenix WinNonlin. Visual predictive checks were performed separately for each final model using 500 simulated datasets. Observed concentration-time data were compared with the simulated 5th, 50th, and 95th percentile profiles.

Goodness-of-fit diagnostics included observed-versus-individual predicted concentration plots (DV vs. IPRED), observed-versus-population predicted concentration plots (DV vs. PRED), conditional weighted residuals versus population predictions (CWRES vs. PRED), and conditional weighted residuals versus time (CWRES vs. IVAR). Model adequacy was assessed by visual inspection of these diagnostic plots and the corresponding VPCs.

### 4.11. Monte Carlo Simulation

Monte Carlo simulations were performed using the final population pharmacokinetic models developed for enrofloxacin and ciprofloxacin in plasma and ISF. Simulations were conducted separately for each analyte and matrix using the NLME module of Phoenix WinNonlin version 8.5.2.4 (Certara USA, Princeton, NJ, USA). For each final model, 5000 virtual individuals were simulated over a 24 h period following administration of a 225 mg oral dose. Simulations incorporated the final population parameter estimates and interindividual variability terms of the respective models. Concentration-time profiles were generated from 0 to 24 h at 0.25 h intervals.

The simulated concentration-time profiles were subsequently subjected to non-compartmental analysis using the same settings as described above. Individual AUC_0–24_ and C_max_ values obtained from the simulated datasets were exported for subsequent pharmacokinetic/pharmacodynamic target attainment analyses.

### 4.12. Probability of Target Attainment Analysis

Probability of target attainment (PTA) analyses were performed separately for enrofloxacin and ciprofloxacin in plasma and ISF. Combined parent-metabolite exposure indices have been applied in previous enrofloxacin PK/PD investigations, either by summing enrofloxacin and ciprofloxacin exposures when similar antibacterial potency was assumed or demonstrated, or by accounting for the relative contribution of the active metabolite [20]. However, construction of a potency-adjusted combined index requires organism-specific and preferably paired enrofloxacin and ciprofloxacin MIC data, together with an assumption regarding the additivity of their antibacterial effects. Because the present study was not focused on a specific bacterial species and no paired enrofloxacin-ciprofloxacin MIC dataset was available, a generally applicable relative-potency weighting factor could not be defined. Enrofloxacin and ciprofloxacin exposures were therefore evaluated independently, and no combined parent-metabolite PK/PD index was calculated.

Individual AUC_0–24_ and C_max_ values obtained from the non-compartmental analysis of the 5000 simulated concentration-time profiles were used to calculate AUC_0–24_/MIC and C_max_/MIC ratios across an MIC range of 0.008–1 μg/mL. This interval encompasses MIC values commonly reported for canine bacterial pathogens and represents the range over which meaningful changes in PTA were observed in the simulations. Across all analyte–matrix combinations, PTA values remained consistently close to 100% below 0.008 μg/mL and became negligible above 1 μg/mL for all evaluated PK/PD targets; therefore, extending the evaluated MIC range would not have provided additional discriminatory information. Plasma PK/PD indices were calculated from measured total plasma concentrations, whereas ISF PK/PD indices were calculated from ultrafiltrate concentrations obtained by in vivo ultrafiltration.

Because the present study was not focused on a specific bacterial species, multiple PK/PD targets reported in the fluoroquinolone literature were evaluated. Target AUC_0–24_/MIC ratios of 30 and 40 were included to reflect exposure thresholds reported for Gram-positive pathogens, whereas target AUC_0–24_/MIC ratios of 100 and 125 and a C_max_/MIC ratio of 8 were included based on commonly applied fluoroquinolone PK/PD targets for Gram-negative pathogens and concentration-dependent antibacterial activity [8,20,39].

For each MIC value and PK/PD target, PTA was calculated as the percentage of simulated individuals achieving or exceeding the predefined target. A PTA of ≥90% was considered indicative of adequate target attainment [20]. PTA calculations were performed in Microsoft Excel (Microsoft Corp., Redmond, WA, USA).

### 4.13. Statistical Analysis

Descriptive statistics were used for the presentation of pharmacokinetic data. Pharmacokinetic parameters obtained from non-compartmental analyses are presented as mean ± standard deviation (SD).

Comparisons between plasma and ISF pharmacokinetic parameters were performed separately for enrofloxacin and ciprofloxacin using paired observations from animals with available data in both matrices. Pharmacokinetic parameters were compared using the Wilcoxon signed-rank test. Statistical significance was defined as *p* < 0.05.

Statistical analyses and graphical data evaluation were performed using Microsoft Excel and Phoenix WinNonlin version 8.5.2.4 (Certara USA, Princeton, NJ, USA).

## 5. Conclusions

Oral administration of enrofloxacin at approximately 20 mg/kg resulted in substantially greater systemic and extracellular exposure than previously reported following conventional dosing regimens in dogs. In particular, interstitial fluid exposure exceeded plasma exposure for the parent compound, highlighting the importance of evaluating antimicrobial disposition at the target site rather than relying exclusively on systemic pharmacokinetics.

Exploratory nonlinear mixed-effects pharmacokinetic modeling adequately described enrofloxacin and ciprofloxacin concentration-time profiles in both plasma and interstitial fluid and enabled exploratory simulation-based PK/PD evaluation that incorporated the pharmacokinetic variability observed within the study population. Across most evaluated PK/PD targets, PTA values were consistently higher in interstitial fluid than in plasma, indicating that assessment of target attainment may differ substantially depending on whether plasma or target-site exposure is considered.

Although the limited number of animals precludes formal breakpoint recommendations, the present findings provide novel evidence that incorporation of interstitial fluid exposure can influence PK/PD target attainment estimates and may have important implications for future fluoroquinolone dose optimization and PK/PD-based susceptibility breakpoint assessment in dogs.

## Figures and Tables

**Table 1:** Non-compartmental pharmacokinetic parameters of enrofloxacin in plasma and interstitial fluid (ISF) after oral administration of enrofloxacin.

Parameter	Unit	Plasma	ISF	*p*-Value
C_max_	µg/mL	2.94 ± 1.39	3.77 ± 2.07	0.3125
T_max_	h	2.88 ± 1.25	4.49 ± 0.82 *	0.0313
AUC_0–24_	µg × h/mL	21.64 ± 15.43	44.93 ± 29.15 *	0.0313
AUC_inf_	µg × h/mL	24.48 ± 18.62	48.84 ± 32.88 *	0.0313
AUC_extrap_	%	12.19 ± 4.49	6.74 ± 4.94	-
AUMC_inf_	µg × h^2^/mL	272.97 ± 268.74	507.97 ± 453.79 *	0.0313
λ_z_	h^−1^	0.13 ± 0.05	0.16 ± 0.06	0.6250
t_1/2_	h	5.89 ± 2.09	4.72 ± 1.46	0.3125
MRT_inf_	h	9.15 ± 4.38	10.07 ± 2.66	0.1250

Footnote: Data are presented as mean ± SD. Plasma pharmacokinetic parameters were calculated from all eight dogs with evaluable plasma data, whereas ISF parameters were calculated from the six dogs with evaluable ISF data. Statistical comparisons between plasma and ISF were performed using the Wilcoxon signed-rank test based exclusively on the six dogs with paired plasma and ISF observations. * Significantly different from plasma (Wilcoxon signed-rank test, *p* < 0.05).

**Table 2:** Non-compartmental pharmacokinetic parameters of ciprofloxacin in plasma and interstitial fluid (ISF) following oral administration of enrofloxacin.

Parameter	Unit	Plasma	ISF	*p*-Value
C_max_	µg/mL	0.37 ± 0.15	0.77 ± 0.34 *	0.0313
T_max_	h	4.50 ± 1.77	8.91 ± 5.25 *	0.0313
AUC_0–24_	µg × h/mL	4.35 ± 1.50	10.68 ± 4.92 *	0.0313
AUC_inf_	µg × h/mL	4.91 ± 2.03	13.18 ± 7.10 *	0.0313
AUMC_inf_	µg × h^2^/mL	62.61 ± 40.27	176.55 ± 117.28	0.0938
AUC_extrap_	%	11.74 ± 9.73	10.80 ± 9.52	-
λ_z_	h^−1^	0.14 ± 0.02	0.14 ± 0.04	0.6875
t_1/2_	h	5.17 ± 0.74	5.12 ± 1.25	0.4375
MRT_inf_	h	11.70 ± 4.40	12.23 ± 3.28	0.2188

Footnote: Data are presented as mean ± SD. Plasma pharmacokinetic parameters were calculated from all eight dogs with evaluable plasma data, whereas ISF parameters were calculated from the six dogs with evaluable ISF data. Statistical comparisons between plasma and ISF were performed using the Wilcoxon signed-rank test based exclusively on the six dogs with paired plasma and ISF observations. * Significantly different from plasma (Wilcoxon signed-rank test, *p* < 0.05).

**Table 3:** Final population pharmacokinetic parameter estimates for enrofloxacin and ciprofloxacin in plasma and interstitial fluid.

Compound	Matrix	Parameter	Estimate	RSE (%)	95% CI
Enrofloxacin	Plasma	K_a_ (h^−1^)	0.78	11.2	0.61–0.96
		CL/F (L × h^−1^)	27.06	32.5	9.50–44.62
		V/F (L)	131.35	17.1	86.66–176.05
		T_lag_ (h)	0.40	31.9	0.15–0.65
	ISF	K_a_ (h^−1^)	0.13	16.3	0.09–0.18
		CL/F (L × h^−1^)	12.10	38.8	2.56–21.64
		V/F (L)	21.70	33.8	6.81–36.60
		T_lag_ (h)	1.47	11.9	1.11–1.82
Ciprofloxacin	Plasma	K_a_ (h^−1^)	0.46	59.1	−0.08–1.00
		CL/F (L × h^−1^)	88.62	29.7	35.96–141.28
		V/F (L)	804.76	40.4	155.47–1454.04
		T_lag_ (h)	0.40	41.0	0.07–0.72
	ISF	K_a_ (h^−1^)	0.24	50.8	−0.01–0.48
		CL/F (L × h^−1^)	34.41	28.0	14.80–54.01
		V/F (L)	334.12	25.4	161.56–506.68
		T_lag_ (h)	1.60	8.7	1.31–1.88

Footnote: K_a_, first-order absorption rate constant; CL/F, apparent clearance; V/F, apparent volume of distribution; T_lag_, absorption lag time; RSE, relative standard error; CI, confidence interval; ISF, interstitial fluid.

**Table 4:** Interindividual variability and shrinkage estimates of the final population pharmacokinetic models.

Compound	Matrix	Parameter	IIV (%CV)	η-Shrinkage (%)
Enrofloxacin	Plasma	V/F	38.8	6.4
		CL/F	113.2	1.2
		T_lag_	111.2	2.7
	ISF	V/F	27.9	27.8
		CL/F	119.2	1.3
Ciprofloxacin	Plasma	V/F	67.1	4.2
		CL/F	79.4	10.7
	ISF	CL/F	72.1	5.3
		K_a_	103.9	4.3

Footnote: Ka, first-order absorption rate constant; CL/F, apparent clearance; V/F, apparent volume of distribution; Tlag, absorption lag time; IIV, interindividual variability, expressed as coefficient of variation. η-shrinkage values are reported for the random effects retained in the final models.

**Table 5:** Probability of target attainment (%) for enrofloxacin in plasma and interstitial fluid (ISF) at different MIC values. Bold values indicate the highest MIC achieving ≥90% PTA.

MIC (µg/mL)	Plasma					ISF				
	C_max_/MIC	AUC_0–24_/MIC				C_max_/MIC	AUC_0–24_/MIC			
	≥8	≥30	≥40	≥100	≥125	≥8	≥30	≥40	≥100	≥125
0.008	100	100	100	99.9	99.7	100	100	100	100	100
0.015	99.9	100	100	99.2	98.6	100	100	100	100	100
0.03	99.4	99.8	99.6	**95.6**	**93.0**	100	100	100	100	100
0.06	**95.6**	98.7	**97.3**	83.2	76.3	100	100	100	100	100
0.125	79.6	**93.0**	87.7	55.7	45.3	**99.1**	100	100	**99.8**	**99.4**
0.25	47.2	76.3	65.3	25.0	17.0	86.0	100	99.9	84.6	59.2
0.5	16.0	47.2	34.1	5.9	3.3	19.5	**99.5**	**95.9**	7.4	1.3
1	2.8	18.4	10.2	0.8	0.3	0	64.7	25.6	0	0

**Table 6:** Probability of target attainment (%) for ciprofloxacin in plasma and interstitial fluid at different MIC values. Bold values indicate the highest MIC achieving ≥90% PTA.

MIC (µg/mL)	Plasma					ISF				
	C_max_/MIC	AUC_0–24_/MIC				C_max_/MIC	AUC_0–24_/MIC			
	≥8	≥30	≥40	≥100	≥125	≥8	≥30	≥40	≥100	≥125
0.008	**97.6**	99.9	99.7	**96.2**	**93.5**	99.5	100	100	99.5	99.2
0.015	89.1	99.1	98.1	78.7	78.7	98.2	99.9	99.8	97.8	**96.2**
0.03	64.0	**94.9**	**90.5**	59.3	48.3	**92.3**	99.4	98.7	**90.5**	85.9
0.06	29.5	80.1	69.1	26.1	17.4	73.4	**96.6**	**93.8**	71.3	62.0
0.125	6.7	48.3	34.4	5.4	2.9	18.7	85.9	77.8	33.8	19.7
0.25	0.7	17.4	9.3	0.6	0.2	0	62.0	47.3	0.5	0
0.5	0	3.3	1.3	0	0	0	22.3	6.4	0	0
1	0	0.3	0.1	0	0	0	0	0	0	0

## Data Availability

The original contributions presented in this study are included in the article/Supplementary Materials. Further inquiries can be directed to the corresponding author.

## Supplementary Materials

The following supporting information can be downloaded at: https://www.mdpi.com/article/10.3390/antibiotics15080716/s1, Table S1: Intra- and inter-day accuracy and precision of enrofloxacin and ciprofloxacin quantification in canine plasma and interstitial fluid. Table S2: Matrix-effect assessment of enrofloxacin and ciprofloxacin in canine plasma and interstitial fluid.
