# Supplementary material for: Exploratory Pharmacokinetic Characterization of Enrofloxacin and Ciprofloxacin in Plasma and Interstitial Fluid of Dogs: Nonlinear Mixed-Effects Modeling and PK/PD Target Attainment Analysis
Source: Antibiotics (Basel). 2026 Jul 23;15(8):716. doi: 10.3390/antibiotics15080716 (PMC13509500; doi:10.3390/antibiotics15080716)
Supplement: Supplementary file 1 [file antibiotics-15-00716-s001.zip › antibiotics-4430983-supplementary.pdf]

Bioanalytical Method Validation

Selectivity

Selectivity was assessed by monitoring the MRM transitions of enrofloxacin and ciprofloxacin in pooled blank plasma and ISF samples. No interfering peaks were observed at the retention times of either analyte in the investigated matrices, demonstrating adequate selectivity of the method for the determination of enrofloxacin and ciprofloxacin.

Calibration curve and quantitation limits

Three independent calibration experiments were performed. Calibration curves were constructed using seven concentration levels over the ranges of 0.5–250 ng/mL for enrofloxacin and 0.05–25 ng/mL for ciprofloxacin. Calibration data were fitted using weighted (1/x) quadratic regression, which provided adequate accuracy and precision across the investigated concentration ranges. A representative weighted (1/x) quadratic regression equation obtained during method validation was $y = -2.02e{-5}x^2 + 24.35x + 2416$ for enrofloxacin and $y = -9.01e{-5}x^2 + 25.04x + 53.64$ for ciprofloxacin. The corresponding coefficients of determination (R^2) were 0.9948 and 0.9955, respectively.

The theoretical lower limits of detection (LLOD) and quantitation (LLOQ), estimated from signal-to-noise ratios, were 6.5 and 16.3 pg/mL, respectively. Identical values were obtained for enrofloxacin and ciprofloxacin because both analytes exhibited comparable detector responses over the investigated concentration range. These values represent theoretical estimates based on signal-to-noise characteristics rather than experimentally verified concentrations.

Although the calculated LLOQ was substantially below the concentration range required for the present study, method performance at low concentrations was experimentally verified at 50 pg/mL. At this concentration, precision, expressed as the coefficient of variation (CV), was below 20%, and accuracy was within ±20% of the nominal concentration, thereby fulfilling the predefined acceptance criteria.

The upper limits of quantitation (ULOQs) were set at 250 ng/mL for enrofloxacin and 25 ng/mL for ciprofloxacin.

Accuracy and precision

Accuracy and precision were assessed using quality control (QC) samples at three concentration levels: 1, 10, and 100 ng/mL for enrofloxacin and 0.1, 1, and 10 ng/mL for ciprofloxacin. Intra-day accuracy and precision were evaluated using three independently prepared replicates at each concentration level. Inter-day performance was assessed over three

independent days using three replicate preparations per concentration level per day (n = 9). The results are summarized in **Supplementary Table S1**.

At all three QC levels (low, medium, and high), intra- and inter-day accuracy and precision met the predefined acceptance criteria. Accuracy was within ±15% of the nominal concentration, and precision, expressed as CV, did not exceed 15%. These results demonstrate adequate repeatability and intermediate precision of the method for the quantitative determination of enrofloxacin and ciprofloxacin in the investigated matrices.

Matrix effect

Matrix effects were evaluated separately in plasma and ISF. Three independent sets of 400-fold concentrated stock solutions were prepared and diluted tenfold with water, plasma, or ISF. Proteins were precipitated by adding three volumes of acetonitrile. After thorough vortex mixing and centrifugation, the supernatants were transferred to Eppendorf tubes and diluted tenfold with ultrapure water to obtain the final analytical solutions.

The extent of ion suppression or enhancement was evaluated by comparing the calculated analyte concentrations in the matrix-containing samples with those obtained for the corresponding water controls. All measured concentrations were within ±15% of the corresponding water controls, indicating that no significant matrix effect was observed under the applied experimental conditions (**Supplementary Table S2**).

Carryover

Carryover was assessed by injecting a blank sample immediately after the highest calibration standard. No detectable analyte peaks were observed in the enrofloxacin or ciprofloxacin MRM channels following injection of the highest calibration standard. These results indicated that no measurable carryover occurred under the applied chromatographic and mass spectrometric conditions.

Stability

Stability was assessed to the extent permitted by the experimental sample-handling conditions. Solid enrofloxacin and ciprofloxacin reference standards stored at 4 °C according to the manufacturers' recommendations were considered stable within their specified storage periods. Stock solutions (1 mg/mL in 1% formic acid) were stored at −20 °C and demonstrated acceptable stability for at least 30 days. Working solutions of enrofloxacin and ciprofloxacin were freshly prepared on a weekly basis.

Because study samples were analyzed shortly after collection and were not subjected to extended storage, a comprehensive long-term stability assessment in plasma and ISF was not

performed. Accordingly, the stability evaluation was limited to the storage conditions and time periods relevant to the analytical workflow used in the present study.

Supplementary Table S1. Intra- and inter-day accuracy and precision of enrofloxacin and ciprofloxacin quantification in canine plasma and interstitial fluid.

CF Plasma						
Nominal concentration [ng/mL]	Intra-day n=3			Inter-day n=9		
	Mean [ng/mL]	RE [ng/mL]	CV [%]	Mean [ng/mL]	RE [ng/mL]	CV [%]
0.1	0.09682	-0.00318	13.5%	0.09441	-0.00559	8.94%
1	0.944	-0.0556	5.18%	0.906	-0.0940	4.44%
10	9.567	-0.433	8.26%	9.289	-0.711	5.67%
CF ISF						
Nominal concentration [ng/mL]	Intra-day n=3			Inter-day n=9		
	Mean [ng/mL]	RE [ng/mL]	CV [%]	Mean [ng/mL]	RE [ng/mL]	CV [%]
0.1	0.09705	-0.00295	2.50%	0.09888	-0.00112	2.60%
1	1.022	0.0221	6.03%	1.039	0.0391	5.54%
10	10.03	0.0321	9.74%	9.990	-0.0104	5.58%
EF Plasma						
Nominal concentration [ng/mL]	Intra-day n=3			Inter-day n=9		
	Mean [ng/mL]	RE [ng/mL]	CV [%]	Mean [ng/mL]	RE [ng/mL]	CV [%]
1	1.009	0.00914	8.49%	0.939	-0.0615	3.51%
10	9.884	-0.116	8.71%	9.886	-0.114	6.48%
100	96.76	-3.24	8.78%	94.57	-5.43	4.86%
EF ISF						
Nominal concentration [ng/mL]	Intra-day n=3			Inter-day n=9		
	Mean [ng/mL]	RE [ng/mL]	CV [%]	Mean [ng/mL]	RE [ng/mL]	CV [%]
1	0.985	-0.0149	6.18%	0.925	-0.0749	5.56%
10	10.17	0.174	9.51%	10.46	0.456	5.02%
100	95.04	-4.96	11.76%	92.50	-7.50	6.13%

Supplementary Table S2. Matrix-effect assessment of enrofloxacin and ciprofloxacin in canine plasma and interstitial fluid.

Matrix CF		
Nominal concentration [ng/mL]	Plasma n=6	ISF n=6
	Recovery [%]	Recovery [%]
0.1	92.6%	95.9%
1	85.5%	95.0%
10	87.2%	91.3%

Matrix EF		
Nominal concentration [ng/mL]	Plasma n=6	ISF n=6
	Recovery [%]	Recovery [%]
1	94.8%	92.8%
10	90.3%	93.2%
100	91.9%	88.1%
